# Emerging therapies for acute myeloid leukemia

**DOI:** 10.1186/s13045-017-0463-6

**Published:** 2017-04-18

**Authors:** Caner Saygin, Hetty E. Carraway

**Affiliations:** 1 Department of Hematology and Oncology, Taussig Cancer Institute, Cleveland Clinic, Cleveland, OH 44195 USA; 2 Department of Hematology and Oncology, Leukemia Program, Taussig Cancer Institute, Cleveland Clinic, Desk R30, Cleveland, OH 44195 USA

**Keywords:** AML, CPX-351, Vosaroxin, Guadecitabine, IDH, HDAC, BET, DOT1L, LSD1, FLT3, Vadastuximab, Volasertib, Venetoclax, Tosedostat

## Abstract

Acute myeloid leukemia (AML) is characterized by clinical and biological heterogeneity. Despite the advances in our understanding of its pathobiology, the chemotherapy-directed management has remained largely unchanged in the past 40 years. However, various novel agents have demonstrated clinical activity, either as single agents (e.g., isocitrate dehydrogenase (IDH) inhibitors, vadastuximab) or in combination with standard induction/consolidation at diagnosis and with salvage regimens at relapse. The classes of agents described in this review include novel cytotoxic chemotherapies (CPX-351 and vosaroxin), epigenetic modifiers (guadecitabine, IDH inhibitors, histone deacetylase (HDAC) inhibitors, bromodomain and extraterminal (BET) inhibitors), FMS-like tyrosine kinase receptor 3 (FLT3) inhibitors, and antibody-drug conjugates (vadastuximab), as well as cell cycle inhibitors (volasertib), B-cell lymphoma 2 (BCL-2) inhibitors, and aminopeptidase inhibitors. These agents are actively undergoing clinical investigation alone or in combination with available chemotherapy.

## Background

Acute myeloid leukemia (AML) is a clonal disorder of myeloid progenitors characterized by clinical and biological heterogeneity. With decades of research, our understanding of the pathobiology, classification, and genomic landscape of AML has improved substantially [1, 2]. Concurrently, various promising agents have been evaluated in clinical trials, but the classical upfront treatment of AML (intensive induction with 7 days of cytarabine plus 3 days of an anthracycline (7 + 3), followed by consolidation chemotherapy or hematopoietic cell transplant (HCT)) has remained steadfast over the last 40 years. With traditional intensive chemotherapy regimens, only 40% of AML patients <60 years of age survive more than 5 years, and even patients with favorable-risk core-binding factor leukemia have a mortality rate of 56% at 10 years [3]. In older adults unfit for standard induction chemotherapy, outcomes of lower-intensity treatment (low-dose cytarabine, azacitidine, or decitabine) are not curative and median overall survival (OS) is often <1 year [4, 5]. This discordance between the explosive growth in trials investigating novel therapies in AML and the minimal progress made in current standard of care might be attributed to suboptimal preclinical models, exclusive criteria that limit patients eligible for enrollment into clinical trials, and single-agent clinical approach to early drug development as well as limited ability to eliminate the remnant leukemic clone due to ineffective novel agents or emergence of leukemic clonal promiscuity [6].

Despite the paucity of new drug approvals for AML treatment, identification of potential driver mutations through next-generation sequencing has revealed biologic intricacies of AML and led to new investigational drugs. Furthermore, antigen-specific immunotherapies hold promise to expand the armamentarium for treatment of AML. In this review, we highlight some of the promising novel approaches and agents that are currently in clinical trials and have published or presented data. This article is not meant to be an exhaustive review of all emerging agents. Instead, we summarize evolving treatment strategies with promising results in AML trials, which hence will likely emerge as new therapeutics in the near future (Table 1).

**Table 1:** Selected emerging therapies for the management of AML

Drug class/mechanism	Agent	Suggested patient population	Single/combination	Phase of development^a^	Ref.
Cytotoxic chemotherapy					
Liposomal formulation of 7 + 3	CPX-351	≥60 years, sAML, fit for induction therapy	Single agent	3	[10–15]
Topoisomerase II inhibitor	Vosaroxin	≥60 years, R/R	With cytarabine	3	[19]
Epigenetic modifiers					
DNMT inhibitor	Guadecitabine	Unfit for intensive therapy or R/R	Single agent	3	[24, 25]
IDH1 inhibitor	AG-120, IDH305, FT-2102	*IDH1* mutated	Single agent/with AZA or induction and consolidation	1b/2	[31, 33]
IDH2 inhibitor	Enasidenib (AG-221)	*IDH2* mutated	Single agent/with AZA or induction and consolidation	3	[34]
HDAC inhibitors	Panobinostat	Ongoing investigation	With HMAs or induction and consolidation	1b/2	[38, 39]
	Vorinostat			3	[40–43]
	Entinostat			2	[45]
	Pracinostat			2	[46]
BET inhibitor	OTX015	Ongoing investigation	Single agent	1	[47]
DOT1L inhibitor	Pinometostat	*MLL*-rearranged	Single agent	1	[49]
LSD1 inhibitor	Tranylcypromine, GSK2879552, ORY-1001	Ongoing investigation	Single agent/with ATRA	1/2	–
FLT3 inhibitors	Sorafenib	*FLT3*-ITD-mutated R/R	With AZA	2	[60]
	Midostaurin	*FLT3*-ITD or *FLT3*-TKD, ≤60 years	With induction and consolidation	3	[63]
	Quizartinib	*FLT3*-ITD-mutated R/R	Single agent	3	[66–68]
	Crenolanib	*FLT3*-ITD or *FLT3*-TKD	Single agent/with induction and consolidation	2	[71–73]
	Gilteritinib	*FLT3*-ITD or *FLT3*-TKD R/R	Single agent	3	[74]
Antibody-drug conjugates					
Anti-CD33	Vadastuximab (SGN-CD33A)	CD33+	Single agent/with HMAs or induction and consolidation	3	[75–77]
Cell cycle inhibitors					
Polo-like kinase inhibitor	Volasertib	Unfit for intensive therapy	With LDAC or decitabine	3	[78]
Other agents					
BCL-2 inhibitor	Venetoclax (ABT-199)	R/R, or older (≥65 years) and unfit for intensive therapy	Single agent/with HMAs or LDAC	2	[82, 83]
Aminopeptidase inhibitor	Tosedostat	≥60 year, R/R or unfit for intensive therapy	Single agent/with cytarabine or HMAs	2	[84–87]

^a^Denotes the furthest phase in development

*7 + 3* 7 days of cytarabine and 3 days of daunorubicin, *AML* acute myeloid leukemia, *AZA* azacitidine, *R/R* relapsed or refractory, *HMA* hypomethylating agent, *LDAC* low-dose cytarabine, *sAML* secondary acute myeloid leukemia

## Novel cytotoxic chemotherapy agents

The backbone of AML induction therapy has been anthracycline-cytarabine combination for decades, and the addition of other cytotoxics, including thioguanine, fludarabine, or etoposide, have offered no additional survival benefit [7, 8]. However, certain modifications in traditional 7 + 3, including manipulation of treatment intensity and duration of treatment, translated into improved OS for selected populations of adult patients [9]. Therefore, new formulations of cytotoxic chemotherapy that have similar mechanisms of action might continue to improve outcomes.

### CPX-351

CPX-351 is the liposomal formulation of cytarabine and daunorubicin packaged at a 5:1 molar ratio within liposomes, which in animal models demonstrated higher efficacy compared with the same drugs administered conventionally [10]. The ratio is shown to be maximally synergistic and minimally antagonistic in vitro [11]. In a randomized, open-label, phase 2 trial, CPX-351 (100 U/m^2^, equivalent to 100 mg/m^2^ cytarabine and 44 mg/m^2^ daunorubicin) was compared with conventional 7 + 3 (100 mg/m^2^ cytarabine and 60 mg/m^2^ daunorubicin) as an induction therapy, with enrollment of 126 AML patients at age 60–75 years, who were fit for intensive chemotherapy [10]. The primary efficacy endpoint was composite complete response (CRc), combining morphologic complete response (CR) and morphologic CR with incomplete count recovery (CRi). Overall, CRc rates were higher (66.7 vs 51.2%, *p* = 0.07) in the CPX-351 arm, which met the predefined criteria for success (*p* < 0.1). There were no differences in true CR rate (48.8% in both arms), event-free survival (EFS), or OS when all patients were analyzed. However, preplanned subgroup analysis of patients with secondary AML (sAML; therapy-related AML or AML with a history of antecedent hematologic disorder) demonstrated an improved response rate (57.6 vs 31.6%, *p* = 0.06), with prolonged EFS (*p* = 0.08) and OS (*p* = 0.01). In a separate phase 2, randomized, open-label study, 125 AML patients at first relapse, who were between the ages of 18 and 65 years, were assigned to CPX-351 or investigators’ choice of first salvage treatment [12]. Overall, the CR rate was slightly higher in the CPX-351 arm (37 vs 31.8%), but the study did not achieve the predefined goal of survival improvement at 1 year.

Based on the promising results in patients with sAML, a randomized, multicenter, open-label, phase 3 study of first-line CPX-351 (100 U/m^2^) vs daunorubicin (60 mg/m^2^) plus cytarabine (100 mg/m^2^) in high-risk sAML patients was initiated [13]. A total of 309 patients between the ages of 60 and 75 years were randomized 1:1 to treatment arms. CPX-351 treatment resulted in better OS (median, 9.56 vs 5.95 months, *p* = 0.005), EFS (*p* = 0.021), and CRc rates (47.7 vs 33.3%, *p* = 0.016). Moreover, 60-day mortality was lower in CPX-351 (13.7 vs 21.2%), and grade 3–5 adverse events (AEs) were similar in frequency and severity in both arms. In a subgroup analysis of this large trial, patients aged 60–69 and 70–75 years were analyzed separately and both groups were found to have greater median OS (9.63 vs 6.87 months in patients aged 60–69 years and 8.87 vs 5.62 months in patients aged 70–75 years) and CRc rate (50 vs 36.3% in patients aged 60–69 years and 43.9 vs 27.8% in patients aged 70–75 years) with the CPX-351 treatment [14]. In another subgroup analysis, 91 transplanted patients were landmarked at the time of hematopoetic stem cell transplant (HSCT) (i.e., time of origin) and patients treated with CPX-351 were found to have better OS as compared to patients treated with standard 7 + 3 (*p* = 0.004) [15]. These results support the use of CPX-351 as first-line induction treatment for fit older patients with sAML, and this formulation may provide an effective bridge to successful HCT in this subset of patients.

In addition, since the drug has shown efficacy (i.e., CRc) at doses of 50 and 75 U/m^2^ in phase 1 and 2 trials, a current phase 2 study compares outcomes of newly diagnosed AML patients at these lower doses, who are otherwise at high risk of induction mortality [16]. Interim results demonstrated significantly better OS (HR, 0.2; *p* = 0.005) and EFS (HR, 0.25; *p* = 0.019) with 75 U/m^2^, and the study continues accrual with the third arm of 100 U/m^2^ (NCT02286726). Moreover, comparison of the outcomes of CPX-351 (100 U/m^2^) vs daunorubicin (90 mg/m^2^) + cytarabine treatment would be valuable to assess the superiority (or non-inferiority) of this formulation.

### Vosaroxin

Vosaroxin is a first-in-class, non-anthracycline quinolone derivative that induces replication-dependent DNA damage by intercalating DNA and inhibiting topoisomerase II, thereby inducing G2 cell cycle arrest and apoptosis [3]. It is minimally metabolized, without production of free radicals that are implicated in the cardiotoxicity observed with other topoisomerase II inhibitors. Based on the encouraging results in early phase 1 and phase 1b/2 studies [17, 18], the randomized, placebo-controlled, double-blind phase 3 VALOR trial of cytarabine (1 g/m^2^ days 1–5) with or without vosaroxin (90 mg/m^2^ days 1–4) was conducted in adult patients with primary refractory AML or AML in first relapse [19]. A total of 711 patients were randomized 1:1 to treatment arms, and the study did not meet the primary endpoint of median OS difference between groups (7.5 months in vosaroxin arm vs 6.1 months in placebo arm, *p* = 0.06). However, the overall CR rate was nearly doubled in the vosaroxin arm compared with the placebo arm (30.1 vs 16.3%, *p* < 0.0001), and the responses were durable as shown by the leukemia-free survival data. Additionally, in the predefined analysis censoring at the time of HSCT, OS was better in the vosaroxin plus cytarabine group than in the placebo plus cytarabine group (6.7 vs 5.3 months, *p* = 0.02). In further preplanned analyses (based on age and time to relapse), vosaroxin-treated patients ≥60 years had significantly better OS (7.1 vs 5 months, *p* = 0.003) and those who relapsed <12 months and received vosaroxin had 1.5 months (6.7 vs 5.2 months, *p* = 0.03) of median OS benefit as compared to the placebo arm. There was no difference in 30- and 60-day all-cause mortality between treatment groups, but 15% of patients on the vosaroxin arm had grade 3 or 4 stomatitis. The recently presented updated survival data was consistent with the subgroup analysis of the primary report, and after a median of 39.9 months of follow-up, the survival benefit observed in patients ≥60 years was durable through 48 months [20].

Vosaroxin has also been investigated in the first-line setting for older patients with previously untreated poor-risk AML. In the single-agent, phase 2 REVEAL-1 trial, vosaroxin monotherapy was evaluated at doses of 72 and 90 mg/m^2^ and the 72 mg/m^2^ dose demonstrated a CRc rate of 35%, with an acceptable 30- and 60-day mortality of 7 and 17%, respectively [21]. Therefore, this dose was used in a subsequent open-label, randomized phase 2 study, which was designed with the “pick a winner” strategy, comparing in a 1:1 randomization of low-dose cytarabine (LDAC) vs vosaroxin monotherapy and LDAC vs LDAC + vosaroxin combination in older unfit AML patients [22]. The study demonstrated no CR or survival benefit for vosaroxin, and the trial was prematurely closed at its first interim analysis.

Vosaroxin at the dose of 90 mg/m^2^ demonstrated clinical activity in combination with cytarabine for relapsed or refractory (R/R) AML patients ≥60 years. However, the OS benefit was 2.1 months. Its utility is limited to the R/R setting, as the drug did not provide benefit when compared to LDAC in the first-line treatment for unfit older patients. A phase 2 study of vosaroxin and decitabine in older patients with newly diagnosed AML or high-risk myelodysplastic syndrome (MDS) is currently ongoing (NCT01893320).

## Epigenetic modifiers

**Fig. 1:**
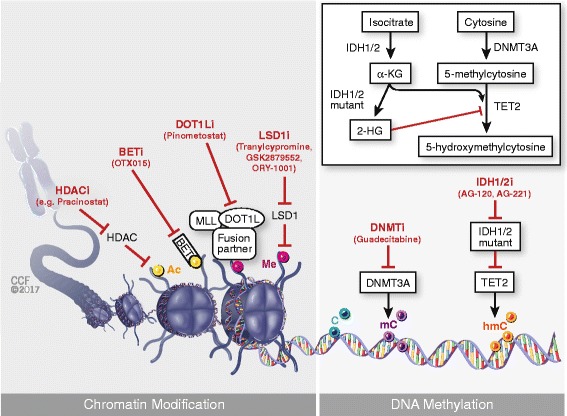
Novel agents targeting epigenetic modifiers in acute myeloid leukemia

A great number of comprehensive whole genome sequencing, exome sequencing, and targeted sequencing studies have been performed in AML and myeloid neoplasms in the last decade. Many of the newly identified recurrently mutated genes are involved in the epigenetic regulation of transcription [1]. Epigenetic modifiers include proteins involved in modifications of DNA cytosine residues (e.g., methylation) or post-translational modifications of histones (acetylation, ubiquitination). Mutations in these genes often lead directly to aberrant gene expression in AML [23]. Currently, these mutations represent a major focus of interest, and several novel epigenetic therapies are in preclinical testing phases or have entered clinical trials (Fig. 1).

### DNMT inhibitors

DNA methyltransferase 3A (*DNMT3A*) catalyzes de novo methylation of cytosine residues in DNA, and its gene is frequently mutated in AML, which leads to loss of function and confers adverse risk. The hypomethylating agents (HMAs), azacitidine and decitabine, are nucleoside analogs that integrate into DNA and inhibit DNMTs [23]. Along with LDAC, they represent a reasonable treatment option for low blast count AML patients who are unfit for intensive induction chemotherapy with CRc rates around 20–30% [4]. Guadecitabine (SGI-110) is a second-generation HMA formulated as a dinucleotide of decitabine and deoxyguanosine, which increases the half-life of decitabine by protecting it from deamination [3]. In a multicenter, phase 2, dose-response study, 51 previously untreated elderly (≥65 years) AML patients, who were ineligible for intensive chemotherapy, were randomized 1:1 to 60 or 90 mg/m^2^ guadecitabine [24]. There were no significant differences in CR rates between treatment groups, and in combined analysis, overall CR and CRc rates were 37 and 57%, respectively, and the median OS was 10.5 months. Based on these results, a randomized phase 3 trial has been initiated to compare guadecitabine vs treatment choice (i.e., azacitidine, decitabine, or LDAC) in patients with previously untreated AML, who are ineligible for intensive chemotherapy (NCT02348489).

Guadecitabine has also been investigated in R/R AML patients, and a recently reported long-term follow-up of phase 2 studies demonstrated that in 103 patients, 23% achieved CRc and median OS was 6.6 months with 1- and 2-year survival rates of 28 and 19%, respectively [25]. On the basis of these data, a phase 3 randomized, open-label study of guadecitabine vs treatment choice in R/R AML has been initiated (NCT02920008).

DNA methylation profiling identified biologically distinct subtypes of AML, and certain methylation profiles were associated with adverse outcome [26, 27]. Furthermore, differentially methylated regions of DNA at baseline distinguished patients who responded to HMA from non-responders in different myeloid malignancies [28, 29]. Specific methylation signatures may predict responsiveness to treatment with guadecitabine and offer an opportunity to improve management of elderly or R/R AML patients.

Guadecitabine has demonstrated clinical activity in first-line and R/R settings, and two ongoing phase 3 studies for these patient populations may provide evidence to justify its use over LDAC and first-generation HMAs. However, a substantial difference in cost with marginal difference in OS benefit might limit its use in the clinical setting.

### IDH inhibitors

**Fig. 2:**
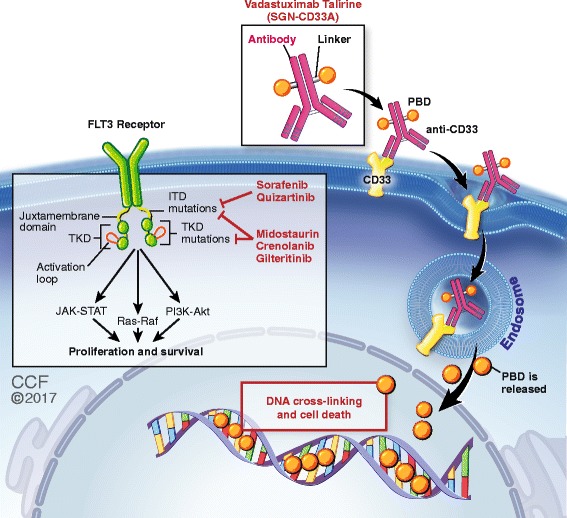
Promising cell surface targets in acute myeloid leukemia

Isocitrate dehydrogenases 1 and 2 (IDH1 (cytoplasmic) and IDH2 (mitochondrial), respectively) catalyze the conversion of isocitrate to α-ketoglutarate (α-KG). In adults, *IDH1* and *IDH2* mutations occur with a frequency of 5–10 and 10–15% in adult AML, respectively, and are more common in patients with cytogenetically normal AML (10.4 and 15–20%, respectively) [23]. All *IDH1* and *IDH2* mutations are novel gain-of-function mutations, and the mutant IDH proteins possess a neomorphic enzyme activity catalyzing the conversion of α-KG to the oncometabolite 2-hydroxyglutarate (2-HG) (Fig. 2). This leads the competitive inhibition of α-KG-dependent enzymes, including TET2, hypermethylation of target genes, and impaired hematopoietic differentiation. The prognostic impact of *IDH* mutations in myeloid neoplasms remains controversial; however, one hypothesis is that inhibition of mutant IDH may decrease the levels of 2-HG and reverse the block in cellular differentiation [30]. In the short time since the *IDH* mutations were first discovered in 2009, several IDH inhibitors have been tested in clinical trials and early results demonstrated encouraging responses with durability and minimal toxicity.

AG-120, IDH305, and FT-2102 are oral inhibitors of mutant IDH1 that are currently in clinical development. In a phase 1, open-label, dose escalation and expansion study, AG-120 monotherapy was evaluated in patients with *IDH1* mutant advanced hematologic malignancies (NCT02074839) [31]. Overall response rate (ORR) was 36% and CR rate was 18% in a cohort in which majority of patients (78%) had R/R AML. The drug was well tolerated, but three patients developed differentiation syndrome within the first 60 days of treatment, and they were successfully managed with hydroxyurea and steroids [32]. Dose expansion arms are currently enrolling patients with R/R and untreated AML. In another phase 1 dose-escalation study, IDH305, a mutant-selective allosteric IDH1 inhibitor, was evaluated in R/R AML and MDS (NCT02381886) [33]. In the interim analysis of 24 AML patients, ORR and CR rates were 33 and 9.5%, respectively. The most commonly reported AEs were raised bilirubin and lipase. A phase 1/1b study of FT-2102 as a single agent and in combination with azacitidine is currently ongoing (NCT02719574).

Enasidenib (AG-221) is an oral inhibitor of IDH2, which also reduces 2-HG levels in patients with *IDH2*-mutated AML to levels detected in healthy subjects [30]. Interim results of a phase 1/2 dose-escalation study of AG-221 demonstrated an ORR of 41% in R/R AML patients, regardless of the number of prior treatments [34]. Rates of CR, CRc, and partial response (PR) were 18, 26, and 15%, respectively. An additional 45% of patients had stable disease. Of interest, the mutant IDH2 variant allelic frequency (VAF) did not change from baseline in the majority of patients who attained CRc, suggesting that eradication of the clone was not necessary for response and gives insight into the putative mechanism of enasidenib as a differentiation agent. The drug was well tolerated, and the most common AEs were indirect hyperbilirubinemia (19%) and nausea (18%). Based on these results, the phase 3, randomized, open-label *IDH*ENTIFY trial is currently recruiting AML patients ≥60 years who are R/R after two or three prior regimens (NCT02577406). Patients are randomized 1:1 to enasidenib or conventional care regimens, and enrollment will continue through 2019.

In early phase 1/2 trials, IDH inhibitors have demonstrated impressive single-agent activity in R/R AML patients. Additionally, both AG-120 and enasidenib are being investigated in patients with newly diagnosed AML with *IDH1* and/or *IDH2* mutations, in combination with induction and consolidation for patients eligible for intensive chemotherapy (NCT02632708), as well as with azacitidine in unfit patients (NCT02677922). A caveat is the lack of OS data, and it is unclear whether patients with “stable disease,” who represent 45% of patients in the initial phase 1/2 trial of enasidenib, will have a meaningful survival benefit. Despite this, these drugs offer a significant possibility of improving current standard of care in *IDH* mutant AML patients.

### HDAC inhibitors

Histone acetylation and deacetylation are essential processes for the regulation of gene expression. Acetylation of histones relaxes the condensed chromatin and exposes the promoter regions of genes to transcription factors. On the other hand, deacetylation catalyzed by histone deacetylases (HDACs) results in gene silencing [23]. In leukemic cells, this balance is disrupted by several mechanisms, and therefore, HDAC inhibitors emerged as an attractive therapeutic approach to modulate disease (Fig. 2). Unlike IDH inhibitors, clinical activity of monotherapy with an HDAC inhibitor was low, with ORR of 17% for vorinostat [35] and 13% for mocetinostat [36], and no clinical response was achieved with entinostat monotherapy [37]. Therefore, current studies focus on combination regimens of HDAC inhibitors with other epigenetic agents like HMAs or alternatively directly intensive chemotherapy.

In a phase 1b/2 study, oral pan-HDAC inhibitor panobinostat was administered sequentially with azacitidine (75 mg/m^2^) to previously untreated patients with AML or high-risk MDS [38]. In 29 patients with AML, CRc and PR rates were 10 and 21%, respectively, and median OS was 8 months. Another phase 1b/2 study, Panobidara, combined panobinostat with induction chemotherapy consisting of idarubicin + cytarabine (IA), followed by panobinostat maintenance in elderly patients with newly diagnosed AML [39]. In 38 evaluable patients, CR was 64% and median OS of the whole cohort was 17 months. Even though results from early clinical studies are encouraging, larger randomized cohorts are necessary to make any statement about the benefit of adding panobinostat to HMAs or intensive chemotherapy.

Despite its modest activity as a single agent, a phase 2 study of vorinostat combined with IA for newly diagnosed young (≤65 years) AML or high-risk MDS patients showed an ORR of 85%, including 76% true CR, and a median OS of 82 weeks [40]. ORR was 93% in diploid patients and 100% in *FLT3* internal tandem duplication (*FLT3*-ITD) patients. Based on these results, the randomized, phase 3 SWOG S1203 study compared 7 + 3 vs IA vs IA plus vorinostat in young (≤60 years) untreated AML patients [41]. There were no significant differences in CR rates, EFS, or OS among all three arms; thus, the vorinostat arm was stopped due to futility. Vorinostat has also been studied in combination with HMAs. In a phase 1 dose-escalation study of vorinostat with decitabine in 29 R/R and 31 untreated AML patients, ORRs with concurrent treatment were 15 and 46%, respectively [42]. Additionally, a recently presented phase 1 study of vorinostat with decitabine in young patients (≤60 years) with R/R AML who had mixed lineage leukemic (*MLL*) partial tandem duplication and received a median of two prior regimens, demonstrated modest toxicity with 35% CRc [43]. The future of vorinostat in patients eligible for intensive chemotherapy is in doubt. However, it has proven clinical activity in combination with HMAs for unfit patients and may find itself an indication for rare biological subsets of AML. Of note, data from the SWOG S1117 study, which randomized 277 high-risk MDS patients to azacitidine vs azacitidine plus vorinostat vs azacitidine plus lenalidomide, demonstrated no significant differences in ORR [44]. The study was not powered to evaluate OS, but a longer follow-up is needed to better evaluate effect on duration of response in patients treated with vorinostat.

Entinostat and pracinostat are two other oral HDAC inhibitors, which are in early phases of development. An open-label phase 2 trial (E1905) randomized 149 patients with AML/MDS to azacitidine (50 mg/m^2^ for 10 days) with or without overlapping/concurrent delivery schedule of entinostat [45]. The addition of entinostat did not increase ORR but was associated with decreased reversal of methylation compared to HMA monotherapy. Therefore, another phase 2 randomized study has opened in order to investigate the activity of prolonged azacitidine combined with two different entinostat schedules (concurrent vs sequential) in elderly (≥60 years) patients with AML (NCT01305499). This study hopes to determine if sequential therapy might improve clinical responses as concurrent administration may cause decreased HMA incorporation due to cell cycle arrest from the overlapping schedule of HDACi. Finally, a randomized phase 2 study that investigated pracinostat combined with azacitidine for elderly (≥65 years) AML patients who were unfit for intensive induction therapy was completed [46]. In 50 evaluable patients, the combination was well tolerated with CR and CRi rates of 32 and 14%, respectively. Most clinical responses occurred within the first 2 cycles and continued to improve with ongoing therapy. Pracinostat was granted Orphan Drug Status by the FDA in 2014, and the follow-up of patients treated on this study continues in order to calculate a median OS.

### BET inhibitors

Bromodomain and extraterminal (BET) proteins play a major role in the epigenetic regulation of gene transcription by binding to acetylated histone tails and recruiting the transcriptional machinery to the promoter regions of genes (Fig. 1). BET inhibitors demonstrated remarkable anti-leukemic activity in vitro and in vivo in various AML models and are currently being tested in multiple early-phase trials [23]. In a dose-escalation, open-label, phase 1 study, orally active BET inhibitor OTX015 was given to 41 older (≥60 years) patients with R/R acute leukemia (36 AML, 1 high-risk MDS) [47]. Two patients achieved CR, one had CRp, and two patients had partial blast clearance. Common AEs were diarrhea and hyperbilirubinemia. The study did not identify any biomarkers to predict response. Various other BET inhibitors have entered early clinical trials in patients with R/R AML, including TEN-010 (NCT02308761), GSK525762 (NCT01943851), and CPI-0610 (NCT02158858).

BET inhibitors have raised great interest as a novel treatment approach, and ongoing phase 1 trials are investigating their single-agent activities. These drugs are also being investigated in combination with standard therapies and other novel agents. Furthermore, a vigorous search for potential biomarkers of response may identify patients with higher likelihood of BET inhibitor response.

### DOT1L inhibitors

Rearrangements of the mixed lineage leukemic (*MLL*) gene at the 11q23 chromosome locus are present in 5–10% of AML cases and portend poor prognosis [48]. Most of the *MLL* fusion partners bind to disruptor of telomeric silencing 1-like (DOT1L), which is postulated to be the oncogenic driver of *MLL*-rearranged (*MLL*-r) AML via its histone methyltransferase activity (Fig. 1). Pinometostat (EPZ-5676) is a DOT1L inhibitor and had robust preclinical activity in *MLL*-r xenograft models [23]. Early results from an open-label phase 1 trial, which enrolled adult R/R acute leukemias, demonstrated an ORR of 12.2% (6 out of 49 patients) with an acceptable safety profile [49]. However, another phase 1 trial conducted in children with R/R *MLL*-r acute leukemia reported no ORR in 18 patients enrolled [50]. Both studies showed evidence of target inhibition, and next steps in development should explore pinometostat combinations with other anti-leukemic agents. Interesting preclinical work demonstrates that NPM1-mutated leukemogenesis is dependent on HOX and MEIS1 expression, which is controlled by specific chromatin regulatory complexes. Inhibition by DOT1L and the menin-MLL pathways can release the block on NPM1-mutated leukemia and result in differentiation. Furthermore, inhibition of the menin-MLL pathway led to profound down regulation of MEIS1 and subsequent suppression of the FLT3 expression [51]. These data suggest that the further development of these agents may ultimately play a large role in NPM1/*FLT3*-ITD-mutated leukemias.

### LSD1 inhibitors

Lysine-specific demethylase 1 (LSD1) is a histone demethylase expressed in leukemic cells and regulates the differentiation block in AML [23, 52, 53]. The enzyme can be targeted by tranylcypromine (TCP), and the combination of ATRA and TCP is currently being investigated in multiple phase 1 and phase 1/2 studies for adults with R/R AML or MDS (NCT02273102, NCT02261779, NCT02717884). Selective TCP derivative LSD1 inhibitors, GSK2879552 and ORY-1001, have also entered early-phase trials for patients with R/R acute leukemia (NCT02177812, EudraCT number 2013-002447-29). Similarly, an open-label phase 1 trial of IMG-7289 with or without ATRA is recruiting participants (NCT02842827). At the time of this writing, no data are available from these studies.

In summary, studies of the mutational landscape of AML highlighted many epigenetic modifiers as attractive targets for personalized therapy. Some of these novel agents showed encouraging clinical activity in early-phase trials. Several other agents are in early preclinical development and will likely be tested in AML trials in the near future (e.g., *EZH2* inhibitors). Utilization of accurate biomarkers of clinical response and rational combinations of targeted agents with other anti-leukemic therapy might revolutionize the management of AML in the near future.

## FLT3 inhibitors

FMS-like tyrosine kinase receptor 3 (*FLT3*) is the most frequently mutated gene in AML. *FLT3* internal tandem duplications (*FLT3*-ITDs) are seen in approximately 25–30% of patients with de novo AML, while an additional 5–10% of patients harbor point mutations in tyrosine kinase domain (TKD) that results in constitutive tyrosine kinase signaling (Fig. 2) [54, 55]. The high frequency of these mutations and the poor prognosis associated with *FLT3*-ITD compel the development of *FLT3* inhibitors. Despite the disappointing results with early FLT3 inhibitors and low single-agent activity, newer agents and combination regimens have yielded encouraging results.

### Sorafenib

Sorafenib is an oral multi-kinase inhibitor with activity against FLT3, KIT, vascular endothelial growth factor receptor (VEGFR), and platelet-derived growth factor receptor (PDGFR). It has been investigated in combination with standard 7 + 3 induction and cytarabine consolidation in elderly patients with AML [56]. The combination did not improve OS or EFS (even in the subgroup analysis of patients with *FLT3*-ITD) but resulted in higher treatment-related mortality. Similarly, combination of sorafenib with azacitidine (for *FLT3*-ITD-mutated AML) or LDAC (for high-risk MDS or AML) did not translate into meaningful survival benefit in untreated elderly patients [57, 58]. However, in a phase 2 trial of intensive chemotherapy (7 + 3 followed by consolidation) with or without sorafenib in younger (≤60 years) untreated AML patients, median EFS was significantly longer in the sorafenib arm vs the placebo arm (21 vs 9 months, *p* = 0.01) [59]. CR rates were similar (60 vs 59%), and the sorafenib group had a higher rate of AEs. In another phase 2 study of azacitidine with sorafenib in 43 patients with R/R AML, 93% had *FLT3*-ITD mutation and the combination resulted in an ORR of 46% [60]. Moreover, sorafenib maintenance after allogeneic HCT was shown to be safe and promising to prevent relapse in early-phase studies of patients with *FLT3*-ITD AML [61].

In first-line management of elderly AML patients, sorafenib demonstrated no benefit. The encouraging results in younger patients need to be supported with long-term OS data. Even though the drug might be useful for treatment of R/R AML, its future is limited due to the era of newer FLT3 inhibitors.

### Midostaurin

Midostaurin is another oral multi-kinase inhibitor of FLT3, KIT, VEGFR, PDGFR, and protein kinase C. In early single-agent studies, activity of midostaurin was limited but promising; hence, it was further investigated in combination with standard intensive chemotherapy. In a phase 1b study of newly diagnosed young (≤60 years) AML patients, a midostaurin 50-mg twice-daily dose schedule in combination with 7 + 3 and high-dose cytarabine (HDAC) post-remission resulted in CR rates of 92 and 74% in patients with *FLT3*-mutant and *FLT3*-wild-type AML, respectively [62]. This led to the development of the international, randomized, placebo-controlled, phase 3 RATIFY trial of midostaurin (50 mg twice daily) in combination with 7 + 3 in young untreated AML patients with *FLT3* mutation (ITD or TKD) [63]. A total of 717 patients were randomized 1:1 to either midostaurin or placebo, and the midostaurin arm had significantly longer median OS (74.7 vs 26 months, *p* = 0.007) and EFS (8 vs 3 months, *p* = 0.004). There were no significant differences in CR (59 vs 54%) and AE rates between the groups. In another phase 1/2 study of midostaurin in combination with azacitidine in R/R AML and MDS, ORR was 26%, while patients with *FLT3*-ITD AML who were previously unexposed to FLT3 inhibitors had an ORR of 33% [64]. Two phase 2 studies are currently investigating midostaurin with azacitidine (NCT01093573) and decitabine (NCT01846624) in *FLT3*-ITD AML.

Addition of midostaurin to first-line treatment has shown remarkable survival benefit in *FLT3*-mutated young AML patients and is expected to reshape their standard of care.

### Quizartinib

Quizartinib is a highly selective FLT3 inhibitor with 10-fold lower affinity for other kinases and the first of its class to achieve meaningful single-agent activity. In a phase 1 study, ORR in R/R AML patients was 17% in the entire cohort and 53% in patients with *FLT3*-ITD mutation [65]. In subsequent phase 2 studies of *FLT3*-ITD-mutated R/R AML, quizartinib monotherapy resulted in CRc and ORR rates of 44–54 and 61–72%, respectively [66–68]. Median duration of response was 3 months. Moreover, a recently presented phase 1/2 study of quizartinib with azacitidine or LDAC in R/R myeloid leukemias demonstrated an ORR of 73% among patients with *FLT3*-ITD, and the median OS was 14.8 months for the entire cohort [69]. A randomized, open-label, phase 3 study of quizartinib monotherapy vs salvage chemotherapy in patients with *FLT3*-ITD-mutated R/R AML is currently recruiting participants (NCT02039726).

Response rates achieved with single-agent quizartinib are highly encouraging, but the short duration of response is of concern. Resistance to quizartinib is attributed to mutations in the TKD of the *FLT3* gene; thus, agents that can overcome this resistance and offer durable remissions may substitute it during drug development and approval.

### Crenolanib

Crenolanib is a selective pan-FLT3 inhibitor (ITD and TKD mutants) and can overcome quizartinib resistance with its activity against the D835 mutant FLT3. Up to 22% of patients develop a TKD mutation during FLT3 inhibitor therapy, but the activity of aforementioned inhibitors against secondary point mutations is limited [70]. In an open-label, single-center, phase 2 study of crenolanib in 38 R/R AML patients with *FLT3*-ITD or *FLT3*-TKD, ORR was 62% in FLT3 inhibitor-naïve patients and 38% in patients who had prior FLT3 inhibitor therapy [71]. Median OS was significantly longer in the former (55 vs 13 weeks, *p* = 0.03). This impressive clinical activity led to another phase 2 trial in which crenolanib in combination with standard 7 + 3 induction and HDAC consolidation was given to newly diagnosed AML patients with *FLT3* mutations [72]. In the interim analysis of 25 patients, the combination was well tolerated and 96% achieved CRc, with 88% true CR. At a median follow-up of 6 months, only three patients (all >60 years) relapsed. OS was not reached, and the trial is ongoing. Finally, interim results of a phase 2 trial of crenolanib in combination with salvage idarubicin and HDAC in multiply R/R *FLT3*-mutated AML showed a CRc rate of 67% from a total of six patients who received ≤2 prior AML therapies, while no one with >2 prior treatments achieved CR [73]. Median OS was longer in the former (259 vs 53 days). This trial is now expanded to allow combination of crenolanib with other salvage regimens.

### Gilteritinib

Gilteritinib is a potent, selective FLT3/AXL inhibitor with activity against both *FLT3*-ITD and *FLT3*-TKD mutations. In a large open-label phase 1/2 study of gilteritinib in R/R AML patients, 252 patients were enrolled and 70% had ≥2 prior AML therapies [74]. While patients with wild-type FLT3 had minimal benefit, ORR in 169 *FLT3*-mutated patients receiving ≥80 mg was 52%, median duration of response was 20 weeks, and median OS was 31 weeks. With these encouraging results, a randomized open-label phase 3 trial of gilteritinib vs salvage chemotherapy in R/R *FLT3*-mutated AML is ongoing (NCT02421939). In addition, a phase 1 study investigating the safety and efficacy of gilteritinib in combination with standard induction and consolidation in newly diagnosed adult patients with AML is ongoing (NCT02236013).

Clinical development of FLT3 inhibitors has been a vigorous effort during the past decade. Newer agents have demonstrated remarkable activity in *FLT3*-mutated R/R AML. However, short durations of response with quizartinib (3 months) and gilteritinib (5 months) can be a limiting factor for patients in whom HCT is not considered. Crenolanib will be an interesting agent to follow due to its reported single-agent activity and is currently also being investigated in combination with other salvage regimens. The RATIFY study has demonstrated benefit of combining midostaurin with standard induction and consolidation therapy of *FLT3*-mutated AML in patients less than 60 years of age, and we await results if this agent will become the first FDA-approved inhibitor in the upfront setting for younger AML patients. In addition, crenolanib was shown to be safe and effective with intensive chemotherapy, and if gilteritinib improves the outcomes of standard therapy in ongoing studies, these two newer agents may help continue the personalized approach to therapy with subsequent incremental improvements in clinical outcome.

## Monoclonal antibodies

Antigen-specific immunotherapies targeting various cell surface proteins on leukemic myeloblasts and leukemic stem cells are in clinical development. Most of these trials, including anti-CD47 (NCT02678338), anti-CD25 (NCT02588092), anti-CD56 (NCT02420873), ipilimumab (i.e., anti-CTLA4) (NCT01757639), and nivolumab (i.e., anti-PD1) (NCT02464657, NCT02397720), have not yet reported any in-depth results; hence, they will not be discussed in this review. Among others, there is considerable interest in the novel antibody-drug conjugate (ADC) vadastuximab talirine (SGN-CD33A).

Vadastuximab is a CD33-directed antibody conjugated to pyrrolobenzodiazepine dimer, and upon binding, the compound is internalized and the dimer is released via proteolytic cleavage in lysosomes, which leads to DNA cross-linking and cell death (Fig. 2). In a phase 1 study of vadastuximab monotherapy in older patients with treatment-naïve CD33+ AML, 54% achieved CRc and ORR was 73% [75]. Another phase 1 study of vadastuximab (10 μg/kg) plus azacitidine or decitabine in untreated older AML patients reported CRc rate of 73%, median RFS of 9.1 months, and 60-day mortality rate of 8% [76]. In both of these studies, grade 4 myelosuppression was the most common AE, but the drug had no off-target AEs. These encouraging results have led to the phase 3 CASCADE trial investigating HMA with or without vadastuximab in older AML patients, which is currently accruing with a goal target of 500 patients (NCT02785900). Moreover, a phase 1b study of vadastuximab in combination with 7 + 3 induction therapy enrolled 42 newly diagnosed young (≤65 years) AML patients and showed acceptable toxicity profile with 78% CRc rate and 88% ORR [77].

Vadastuximab has remarkable clinical activity with no off-target toxicity as a single agent and with HMAs in elderly patients. If the results of CASCADE trial demonstrate survival benefit, it may improve the current standard of care for older adults who are ineligible for induction chemotherapy.

## Cell cycle inhibitors

Inhibitors of cyclin-dependent kinases, Wee1, MDM2, aurora kinases, and polo-like kinases (PLKs) have been investigated in an effort to block the proliferation of leukemic myeloblasts. However, most of these agents showed little to no additional benefit to standard of care, and the most encouraging results have come from the PLK-inhibitor volasertib.

PLKs play an important role in many cellular processes including entry into mitosis, DNA replication, and stress response to DNA damage. PLK1 is overexpressed in AML cells, and its inhibition with volasertib leads to disrupted spindle formation and cell cycle arrest. In a phase 2 trial, 89 previously untreated AML patients who were unfit for intensive chemotherapy were randomized 1:1 to LDAC with or without volasertib [78]. The LDAC + volasertib arm had a higher CRc rate (31 vs 13.3%, *p* = 0.05), longer median EFS (5.6 vs 2.3 months, *p* = 0.02), and OS (8 vs 5.2 months, *p* = 0.04). A randomized, placebo-controlled, double-blind, phase 3 trial is ongoing to validate the efficacy and safety of this combination (NCT01721876). Combination of volasertib with decitabine in older AML patients is also under investigation (NCT02003573).

## Other agents

### BCL-2 inhibitors

Overexpression of the anti-apoptotic protein B-cell lymphoma 2 (BCL-2) has been implicated in AML cell survival and chemotherapy resistance [79]. Venetoclax is an orally bioavailable, selective BCL-2 inhibitor and showed promising preclinical activity in AML xenograft models [80]. In a single-arm phase 2 study, venetoclax monotherapy resulted in one CR and four CRs out of 28 patients evaluable, majority of whom had R/R AML [81]. Of note, 33% of patients with *IDH1/2* mutations achieved CRc, which might suggest BCL-2 dependence in this subgroup. Despite its modest single-agent activity, venetoclax combined with LDAC or HMAs showed encouraging results in newly diagnosed older (≥65 years) AML patients who were ineligible for intensive therapy. In a phase 1b/2 study of 18 patients, venetoclax + LDAC demonstrated an ORR of 44% with acceptable tolerability [82]. Another phase 1b study evaluated 39 patients treated with venetoclax plus decitabine or azacitidine and showed an ORR of 76% (82% in *IDH1/2*-mutated patients) [83]. These initial findings demonstrate acceptable toxicity and promising clinical activity with venetoclax combinations in older untreated AML patients and warrant future randomized studies to further investigate the efficacy and biomarkers of response.

### Aminopeptidase inhibitors

Aminopeptidases regulate protein turnover by hydrolyzing the terminal amino acids from peptides, which is thought to be an important source of amino acids for malignant cells. Tosedostat is an oral aminopeptidase inhibitor, which showed significant anti-leukemic activity as a single agent, with an ORR of 27% in 51 AML patients, majority of whom were R/R [84]. In the open-label phase 2 OPAL study, 76 older patients (≥60 years) with R/R AML were randomized 1:1 to two different doses of tosedostat (120 mg once daily for 6 months or 240 mg once daily for 2 months followed by 120 mg for 4 months) and the study reported an overall CRc rate of 10% [85]. Efficacy of tosedostat in combination with cytarabine or azacitidine was investigated in a phase 1/2 study of R/R AML or high-risk MDS patients and showed an ORR of 33% [86]. Finally, a phase 2 study of tosedostat with cytarabine or decitabine in newly diagnosed older patients with AML or high-risk MDS demonstrated a CRc rate of 53% and true CR rate of 41% [87]. The combination was well tolerated, and median OS was 11.5 months.

### JAK/STAT inhibitors

JAK2 mutations or fusion proteins leading to constitutive activation of JAK2 have been implicated in myeloproliferative neoplasms and AML. An oral JAK2 inhibitor, pacritinib (SB1518), was shown to have a synergistic effect with pracinostat in preclinical AML models [88]. A phase 2 study of pacritinib in combination with decitabine or cytarabine in older AML patients is currently ongoing (NCT02532010).

## Conclusions

AML is a complex heterogeneous disease with multiple recurrently mutated genes. With increased understanding of AML pathogenesis, there is a strong impetus for development of novel agents against various actionable targets. Some of these agents, like IDH inhibitors, demonstrated impressive single-agent activity, but rational combinations can offer the greatest benefit as far as the diverse genetic landscape is concerned. A decade from now, it is possible that CPX-351 may be added to the standard first-line induction therapy for older adults with secondary AML depending on the cost/benefit ratio. Additionally, novel agents like IDH inhibitors, FLT3 inhibitors, and vadastuximab (SGN-CD33A) may routinely be combined with traditional induction and consolidation therapy in select patients. Improvements in outcomes in R/R AML patients may be realized by combining standard salvage regimens with vosaroxin, guadecitabine, venetoclax, and perhaps even with tosedostat. Furthermore, efforts to boost the host immune system for improved tumor surveillance and killing by the addition of checkpoint inhibitors (ipilimumab, nivolumab and others) may change the delivery of therapy at several phases including intensive chemotherapy, and at the time of relapse, and in the post-HCT setting. Timely application of targeted therapies (e.g., FLT3 inhibitors) in the setting of post-transplant maintenance may also drive improvements for leukemic patients. Development of drugs for AML is a challenging task with a long history of multiple failures and minimal gains. However, in this genomic age with various promising agents, one should have no hesitation to expect a revolution.

## Abbreviations

ATRA: All-trans retinoic acid
NPM: Nucleophosmin
